# Supplementary material for: Arrayed in vivo barcoding for multiplexed sequence verification of plasmid DNA and demultiplexing of pooled libraries
Source: Nucleic Acids Res. 2024 May 6;52(10):e47. doi: 10.1093/nar/gkae332 (PMC11162764; doi:10.1093/nar/gkae332)
Supplement: gkae332_Supplemental_Files [file gkae332_supplemental_files.zip › Supplementary Tables S3, S4, S5, S7.pdf]

Supplemental Tables

Table S1. Cost estimates on sequence verification, high throughput *

Table S2. Cost estimates on sequence verification, low throughput *

Table S3. Bacterial strains used in this study

Table S4: Plasmids used in *in vivo* barcoding

Table S5. DNA oligonucleotides used in this study

Table S6. Sequences for 1,100 oligonucleotides randomly sampled from the human reference genome *

Table S7. The composition of gap filling mix used for capturing *E. coli* ORFs

Table S8. LASSO probes used in this study *

Table S9. Oligonucleotides used for Illumina sequencing *

(* Tables S1, S2, S6, S8 and S9 are included in other uploaded .xls files.)

Table S3. Bacterial strains used in this study

Strains	Genotype	Source
BUN20	[Δlac-169 rpoS(Am) robA1 creC510 hsdR514 ΔuidA(MluI):pir-116 endA(BT333) recA1 F'(lac+ pro+ ΔoriT:tet)]	MAGIC (12)
BW23474	[Δlac-169 rpoS(Am) robA1 creC510 hsdR514 ΔuidA(MluI):pir-116 endA(BT333) recA1]	Gift from Barry Wanner
BW28705	[lacI^Q rrnB3 ΔlacZ4787 hsdR514 Δ (araBAD)567 Δ (rhaBAD)568 galU95 ΔendA9:FRT ΔrecA635:FRT]	Gift from Barry Wanner

Table S4: Plasmids used in this study

Name	Plasmid Type	Homology Regions	Swapping cassette	Other features	Source
pSL361	Helper	NA	NA	pSC101 ori, P_{rhaBAD}-I-SceI, P_{lac}-red, recA	this paper
pSL937	Recipient	HU, HD	P_{rhaBAD}-relE	pBR322 ori, GmR	this paper
pSL438	Donor	HU, HD	HygR-SacB	oriT, R6K oriγ, KanR	this paper
pSL438_BC	Donor	HU, HD	HygR-SacB	oriT, R6K oriγ, KanR, 15-nt barcodes	this paper
pSL439	Donor	HU, HD	HygR-SacB	oriT, R6K oriγ, KanR	this paper
pSL439_BC	Donor	HU, HD	HygR-SacB	oriT, R6K oriγ, KanR, 15-nt barcodes	this paper
pSL1071	Donor	HU, HD	NsrR-PheS	oriT, R6K oriγ, KanR	this paper
pSL1064	Donor	HU, HD	HygR-SacB	oriT, R6K oriγ, KanR	this paper
pML104	Helper	NA	NA	pSC101 ori, P_{lac}-red, recA, SpeR	MAGIC (12)

pSLC-217	Other	NA	NA	R6K oriγ, P_{rhaBAD}-relE	Addgene (14)

Table S5. DNA oligonucleotides used in this study

Name	Sequences	Use
pXL633	CCAGCTGCGGCCGCNNNNNNAANNNNNTTNNNNNGCCATGCATATGGGTTACCT	an oligonucleotide library containing donor barcodes
pXL585	AGCAGTGCGGTAGTAAAGGT	paired with pXL633 to generate the donor barcode library
pXL583	TTGAATGCACCAAAAACTCG	forward primer for amplifying regions from pSL439_BC and pSL438_BC that contain donor barcodes
pXL584	TATCAGTAACAAACCCGCGC	reverse primer for amplifying regions from pSL439_BC and pSL438_BC that contain donor barcodes
pXL631	CCAGCTCTCGAGNNNNNAANNNNNTTNNNNNTTNNNNNTGTCACGCTTACATTCACG	an oligonucleotide library containing recipient barcodes
pXL154	TTTTTGTGATGCTCGTCAGG	paired with pXL631 to generate the recipient barcode library

oSL1581	AGGCGCGCCACCGCTAAGCTCAAGGTCACA	forward primer for amplifying the *E. coli* ORF library and introducing AscI recognition site
oSL1582	TAGCGGCCGCCTTCCGTACCAGGAGAAGGG	reverse primer for amplifying the *E. coli* ORF library and introducing NotI recognition site
AttB1CaptF	GGGGACAAGTTTGTACAAAAAAGCAGGCTTCACCGCTAAGCTCAAGGTCACA	forward primer for post-capture PCR of *E. coli* ORFs captured by LASSO probes
AttB1CaptR	GGGGACCACTTTGTACAAGAAAGCTGGGTCCTAATCTTCCGTACCAGGAGAAGGG	reverse primer for post-capture PCR of *E. coli* ORFs captured by LASSO probes
skpp-101-F	GCTTATTCGTGCCGTGTTAT	forward primer for amplifying 300-nt oligonucleotide pools (25)
skpp-101-R	TACTTTTGATTGCTGTGCCC	reverse primer for amplifying 300-nt oligonucleotide pools (25)

Table S7. The composition of gap filling mix used for capturing *E. coli* ORFs

Component	Volume for 100 μL
PCR grade water	73.8 μL
Glycerol	10 μL
10 mM dNTPs	0.4 μL
Ampligase DNA Ligase (100 U μ l−1)	1 μL
Kapa HiFi	0.8 μL
β-Nicotinamide adenine dinucleotide (NAD+) 50mM	4 μL
10× Ampligase DNA Ligase Buffer	10 μL
